# Unusual magnetotransport in twisted bilayer graphene

**DOI:** 10.1073/pnas.2118482119

**Published:** 2022-04-11

**Authors:** Joe Finney, Aaron L. Sharpe, Eli J. Fox, Connie L. Hsueh, Daniel E. Parker, Matthew Yankowitz, Shaowen Chen, Kenji Watanabe, Takashi Taniguchi, Cory R. Dean, Ashvin Vishwanath, M. A. Kastner, David Goldhaber-Gordon

**Affiliations:** ^a^Department of Physics, Stanford University, Stanford, CA 94305;; ^b^Stanford Institute for Materials and Energy Sciences, SLAC National Accelerator Laboratory, Menlo Park, CA 94025;; ^c^Department of Applied Physics, Stanford University, Stanford, CA 94305;; ^d^Department of Physics, Harvard University, Cambridge, MA 02138;; ^e^Department of Physics, University of Washington, Seattle, WA 98195;; ^f^Department of Materials Science and Engineering, University of Washington, Seattle, WA 98195;; ^g^Department of Physics, Columbia University, New York, NY 10027;; ^h^Department of Applied Physics and Applied Mathematics, Columbia University, New York, NY 10027;; ^i^Research Center for Functional Materials, National Institute for Materials Science, Tsukuba 305-0044, Japan;; ^j^International Center for Materials Nanoarchitectonics, National Institute for Materials Science, Tsukuba 305-0044, Japan;; ^k^Department of Physics, Massachusetts Institute of Technology, Cambridge, MA 02139

**Keywords:** Hofstadter’s butterfly, twisted bilayer graphene, anisotropy

## Abstract

We present transport measurements of bilayer graphene with a 1.38^∘^ interlayer twist. As with other devices with twist angles substantially larger than the magic angle of 1.1^∘^, we do not observe correlated insulating states or band reorganization. However, we do observe several highly unusual behaviors in magnetotransport. For a large range of densities around half filling of the moiré bands, magnetoresistance is large and quadratic. Over these same densities, the magnetoresistance minima corresponding to gaps between Landau levels split and bend as a function of density and field. We reproduce the same splitting and bending behavior in a simple tight-binding model of Hofstadter’s butterfly on a triangular lattice with anisotropic hopping terms. These features appear to be a generic class of experimental manifestations of Hofstadter’s butterfly and may provide insight into the emergent states of twisted bilayer graphene.

## Significance

When two sheets of graphene are twisted to the magic angle of 1.1^∘^, the resulting flat moiré bands can host exotic correlated electronic states such as superconductivity and ferromagnetism. Here, we show transport properties of a twisted bilayer graphene device at 1.38^∘^, far enough above the magic angle that we do not expect exotic correlated states. Instead, we see several unusual behaviors in the device’s resistivity upon tuning both charge carrier density and perpendicular magnetic field. We can reproduce these behaviors with a surprisingly simple model based on Hofstadter’s butterfly. These results shed light on the underlying properties of twisted bilayer graphene.

The mesmerizing Hofstadter butterfly spectrum arises when electrons in a two-dimensional periodic potential are immersed in an out-of-plane magnetic field. When the magnetic flux Φ through a unit cell is a rational multiple *p* / *q* of the magnetic flux quantum $\Phi_{0}=h/e$, each Bloch band splits into *q* subbands (1). The carrier densities corresponding to gaps between these subbands follow straight lines when plotted as a function of normalized density $n/n_{s}$ and magnetic field (2). Here, *n_s_* is the density of carriers required to fill the (possibly degenerate) Bloch band. These lines can be described by the Diophantine equation $(n/ns)=t(\Phi /\Phi_{0})+s$ for integers *s* and *t*. In experiments, they appear as minima or zeros in longitudinal resistivity coinciding with Hall conductivity quantized at $\sigma_{xy}=te^{2}/h$ (3, 4). Hofstadter originally studied magnetosubbands emerging from a single Bloch band on a square lattice. In the following decades, other authors considered different lattices (5–7), the effect of anisotropy (6, 8–10), next-nearest-neighbor hopping (11–15), interactions (16, 17), density wave states (9), and graphene moirés (18, 19).

It took considerable ingenuity to realize clean systems with unit cells large enough to allow conventional superconducting magnets to reach $\Phi /\Phi_{0}∼1$. The first successful observation of the butterfly in electrical transport measurements was in GaAs/AlGaAs heterostructures with lithographically defined periodic potentials (20–22). These experiments demonstrated the expected quantized Hall conductance in a few of the largest magnetosubband gaps. In 2013, three groups mapped out the full butterfly spectrum in both density and field in heterostructures based on monolayer (23, 24) and bilayer (25) graphene. In all three cases, the authors made use of the 2% lattice mismatch between their graphene and its encapsulating hexagonal boron nitride (hBN) dielectric. With these layers rotationally aligned, the resulting moiré pattern was large enough in area that gated structures studied in available high-field magnets could simultaneously approach normalized carrier densities and magnetic flux ratios of 1. Later work on hBN-aligned bilayer graphene showed that, likely because of electron–electron interactions, the gaps could also follow lines described by fractional *s* and *t* (26).

In twisted bilayer graphene (TBG), a slight interlayer rotation creates a similar-scale moiré pattern. Unlike with graphene–hBN moirés, in TBG there is a gap between lowest and neighboring moiré subbands (27). As the twist angle approaches the magic angle of 1.1^∘^ the isolated moiré bands become flat (28, 29), and strong correlations lead to fascinating insulating (30–37), superconducting (31–33, 35–37), and magnetic (34, 35, 38) states. The strong correlations tend to cause moiré subbands within a fourfold degenerate manifold to move relative to each other as one tunes the density, leading to Landau levels that project only toward higher magnitude of density from charge neutrality and integer filling factors (37, 39). This correlated behavior obscures the single-particle Hofstadter physics that would otherwise be present.

In this work, we present measurements from a TBG device twisted to 1.38^∘^. When we apply a perpendicular magnetic field, a complicated and beautiful fan diagram emerges. In a broad range of densities on either side of charge neutrality, the device displays large, quadratic magnetoresistance. Within the magnetoresistance regions, each Landau level associated with ν=±8,±12,±16,… appears to split into a pair, and these pairs follow complicated paths in field and density, very different from those predicted by the usual Diophantine equation. Phenomenology similar in all qualitative respects appears in measurements on several regions of this same device with similar twist angles and in two separate devices, one at 1.59^∘^ and the other at 1.70^∘^ (see *SI Appendix* for details).

We reproduce the unusual features of the Landau levels (LLs) in a simple tight-binding model on a triangular lattice with anisotropy and a small energetic splitting between two species of fermions. At first glance, this is surprising, because that model does not represent the symmetries of the experimental moiré structure. We speculate that the unusual LL features we experimentally observe can generically emerge from spectra of Hofstadter models that include the same ingredients we added to the triangular lattice model. With further theoretical work it may be possible to use our measurements to gain insight into the underlying Hamiltonian of TBG near the magic angle.

## Measurements

We fabricated this TBG device using the “tear-and-stack” dry transfer method along with standard lithographic techniques (27, 40). We encapsulated the device in hBN and included both a graphite back gate and a Ti/Au top gate. Using both gates, we could independently tune density *n* and perpendicular displacement field *D* (41).

When stacking, we attempted to crystallographically align the top layer of graphene to the top layer of hBN. Based on optical micrographs taken during stacking, we appear to have succeeded to $∼1.5\pm 0.5^{{}^{\circ}}$. This alignment may have modified the single-particle band structure by breaking sublattice symmetry. Near the magic angle, this can result in a quantum anomalous Hall effect, perhaps particularly when the graphene–graphene and graphene–hBN moiré patterns are commensurate (42). We did not see any features in transport clearly associated with the hBN alignment, so we do not know what role such alignment played, if any. In fact, the alignment of facets that we observed visually may have been between zigzag in one material and armchair in the other, in which case the effect of the hBN on the graphene electronic structure may be much weaker. The two other devices that displayed similar magnetotransport, described in *SI Appendix*, did not appear to have aligned hBN.

**Fig. 1.**
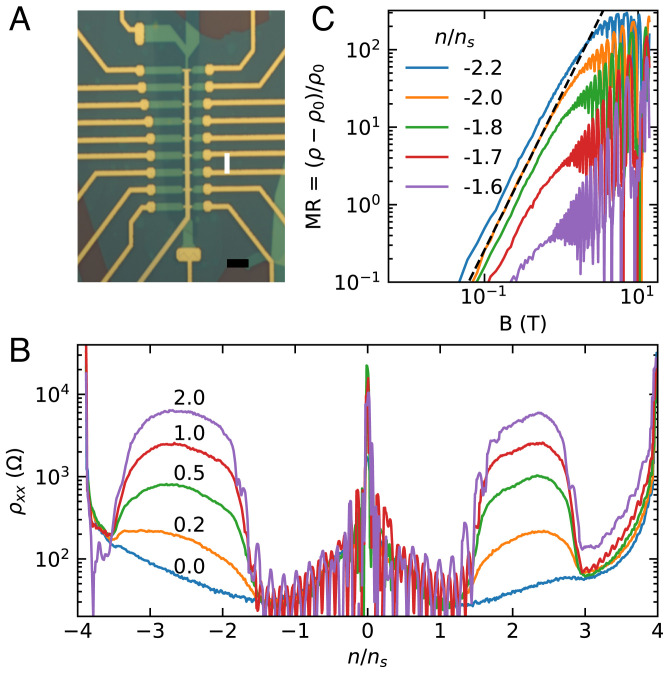
Low-field magnetotransport. (*A*) Optical micrograph of the device showing contacts and top gate in gold and hBN in green. We use the large top and bottom contacts to source and drain current. The channel width is 1 µm, and all longitudinal contact pairs are separated by three squares. The white line indicates the contact pair that we study throughout this work. (Scale bar: 5 µm.) (*B*) Longitudinal resistivity of the device as density is tuned through empty to full moiré cell at several fixed magnetic fields (in Tesla). The peak at *n* = 0 is charge neutrality, and the peaks at the edges of the plot are full filling/emptying of the moiré unit cell. At nonzero fields, there are regions on either side of charge neutrality with large, positive magnetoresistance. (*C*) Magnetoresistance ratio as a function of field for several fixed densities on a log-log plot. Each trace is offset vertically for clarity. The black dashed line is a quadratic.

Fig. 1*A* shows an optical micrograph of the completed device: a standard Hall bar with nine voltage probes on each side. The conduction channel is 1-µ m wide and all contact pairs are separated by three squares. In this work, we focus on measurements from only one contact pair with twist angle 1.38^∘^ ± 0.01^∘^; however, *SI Appendix* has more information on the other contact pairs. In summary, the twist angle for most contact pairs varies between 1.29^∘^ and 1.45^∘^, with the magnetotransport effects that are the focus of this work being peaked around 1.36^∘^. Curiously, two sets of contact pairs near 1.33^∘^ display relatively mild unusual magnetotransport behaviors, but also display superconductivity (see *SI Appendix* for details). This is far outside the range of twists around the magic angle where superconductivity has been previously reported for twisted bilayer graphene.

Upon tuning the top gate at fixed magnetic field, we do not observe correlated insulating states at partial fillings of the flat bands (Fig. 1*B*). This behavior is consistent with reports of samples similarly far above the magic angle (33). Nor do we observe the opening of a gap at charge neutrality or any signatures of ferromagnetism, behaviors that are associated with aligned hBN near the magic angle (34, 35, 38). Instead, in a broad range of densities near half filling, we observe large positive magnetoresistance for both electron and hole doping. The magnetoresistance ratio [ρ(B)−ρ(0)]/ρ(0) is approximately quadratic at low field, reaches over 300, and appears to saturate above 5 T (Fig. 1*C*).

**Fig. 2.**
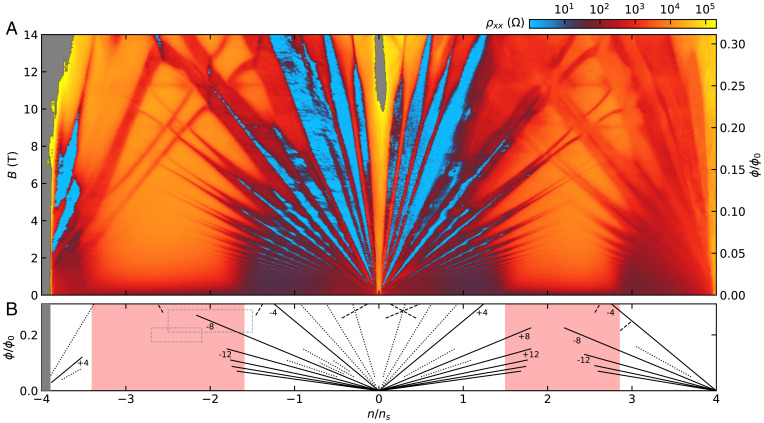
Unusual Landau fan diagram. (*A*) Landau fan diagram taken at 26 mK. Landau-level gaps are observed as minima in longitudinal resistivity. (*B*) Schematic fan diagram corresponding to *A*. Red shaded regions are regions with large magnetoresistance at low field. Solid (dotted) lines are symmetry-preserving (-broken) LLs coming from either charge neutrality or a band edge. Dashed lines are resistance minima corresponding to nonzero *s* and *t*. The light gray dashed boxes indicate regions reproduced in Fig. 3.

As we tune both field and density, a complicated series of quantum oscillations originates at the charge neutrality point and *B* = 0 and propagates outward (Fig. 2*A*). Near charge neutrality, the Landau levels look similar to those of ordinary magic-angle TBG devices (31, 33, 36), with filling fractions ν=±4,±8,±12,… being the most prominent. To within experimental precision, these have zero longitudinal resistance and quantized Hall resistance. As we tune the density into the regions with large magnetoresistance, the Landau levels ν=±6,±10,… disappear. Each fourfold degenerate Landau level appears to split into a pair with slopes roughly corresponding to ν=±8±0.5,±12±0.5, and so on (our field range does not allow tracking the ν=±4 levels into the magnetoresistance regions). These split levels do not have zero longitudinal resistance, reaching a minimum of a few hundred ohms. Nor do they follow exactly straight lines. Instead, they bend when approaching other levels. For lack of a better term, we continue to refer to them as Landau levels.

Landau levels also propagate inward from full filling/emptying of the isolated moiré bands toward lower electron/hole filling, respectively, and these behave similarly to those originating from charge neutrality. We can determine $\Phi /\Phi_{0}$ by considering the points where these levels cross those originating at charge neutrality. For instance, the level with ν=+8 originating at $n/n_{s}=-4$ must intersect the level with ν=−12 originating at charge neutrality at $\Phi /\Phi_{0}=1/5$. In the following *Discussion*, we refer to fields by their values of $\Phi /\Phi_{0}$.

**Fig. 3.**
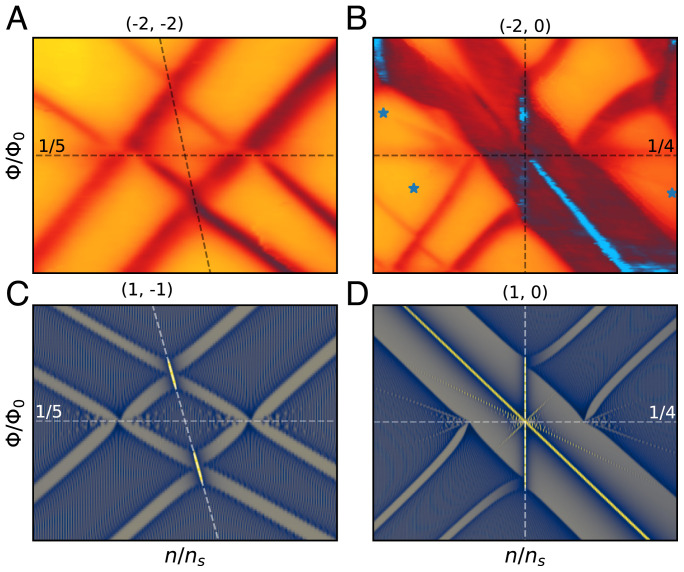
Split Landau-level overlap behavior in experiment and computation. (*A* and *B*) Detail of the crossing of split LLs –12 from charge neutrality (*s*, *t* = 0, –12) and + 8 from $n/n_{s}=-4$ (*s*, *t* = –4, 8) (*A*) and –8 from charge neutrality and + 8 from $n/n_{s}=-4$ (*B*). The horizontal lines are at the indicated $\Phi /\Phi_{0}$, and the lines with steep slopes are the average (*s*, *t*) of the crossing LLs. For the case of *A*, this is the average of (0, –12) and (–4, 8), which is (–2, –2) as indicated. (*C* and *D*) Computed inverse density of states for $q=2,999,\;a_{1}=3,\;a_{2}=a_{3}=1$, and *V* = 0.3 near the crossing of the split levels *s*, *t* = 2, –6, and 0, 4 (*C*) and *s*, *t* = 0, 4, and 2, –4 (*D*). Note that if we were to add in an additional factor of 2 to *s* and *t*, to account for an additional degeneracy, then the values of *s* and *t* in *C* and *D* would match those in *A* and *B* after shifting the zero point of the density. The color scale for *C* and *D* is as in Fig. 4. Stars in *B* indicate the ends of the faint “extra” LLs originating from the intersections of lower (upper) with upper (lower) split LLs. These features are clearly reproduced in computation in *D*.

The phenomenology near the intersection of split Landau levels traveling in opposite directions follows a consistent pattern throughout the fan diagram. The example of the + 8 and –12 levels from the previous paragraph is shown in Fig. 3*A*. As mentioned above, each Landau level splits into a lower and an upper level. When a lower (upper) level overlaps with a lower (upper) level moving in the other direction, it changes direction to follow a line originating from half filling (s=±2, steeply sloped dashed lines in Fig. 3) with slope equal to the average of the two intersecting levels, which is –2 in this case. Within the overlap, the resistivity minima tend to be deeper. Two crossings of a lower with an upper level occur at the same field that the nonsplit Landau levels would have intersected (horizontal dashed lines in Fig. 3), which is 1/5 for this example. There is no drop in resistivity where these two intersect. Instead, they appear to displace horizontally by the width of the level that they are crossing before resuming their previous slope. The result of these changes in direction is that in between the overlaps the split LLs are shifted slightly toward each other.

The overlap of the split LLs around + 8 and –8 originating from $n/n_{s}=-4$ and charge neutrality, respectively, shows the same phenomena (Fig. 3*B*). In this case, the intersection is at $\Phi /\Phi_{0}=1/4$, and the average slope is 0, so we see a vertical line of low resistivity. In addition, there are faint additional levels emanating outward from the two intersections of lower with upper levels.

## Discussion

Surprisingly, we find that we can reproduce the basic phenomenology observed in the Landau fan diagram with a single-particle calculation that is a simple extension of Hofstadter’s butterfly. Rather than attempting to augment the standard continuum model of twisted bilayer graphene (18, 43), we make our calculation in a simple triangular lattice. We do not expect that the exact details of our calculation match the details in our TBG device, including but not limited to the degeneracies arising from spin and valley. Instead, the replication of several distinctive behaviors in such a simple model suggests that they are generic features of Hofstadter-like models. We have verified that the same phenomena arise on a square lattice as well as a honeycomb lattice with modifications equivalent to those described below.

Hofstadter’s butterfly is the result of applying a magnetic field to the tight-binding Hamiltonian

[1] $$H=\sum_{\langle i,j\rangle }a_{ij}c_{i}^{†}c_{j}+\text{h}.\text{c}.$$

via Peierls substitution, where i,j∈ℤ index lattice sites. Each unit cell contains three bonds with associated hopping amplitudes *a*_1_, *a*_2_, and *a*_3_. Following many prior works, we numerically solve the associated eigenvalue equation to find the energy spectrum. We then make the simple step of displaying dμ/dn, the inverse density of states as a function of density. As we show below, if we slightly modify Hofstadter’s tight-binding Hamiltonian, dμ/dn emulates the striking phenomenology of our device’s magnetoresistance.

Specifically, we augment the Hamiltonian by allowing the hopping amplitudes to be different along the three symmetric directions (*a*_1_, *a*_2_, and *a*_3_ not all equal) and adding a second fermion species with a tunable energy splitting *V*, yielding

[2] $$\begin{array}{l} H=\;\sum_{\alpha \in \{A,B\}}\sum_{\langle i,j\rangle }a_{ij}c_{i\alpha }^{†}c_{j\alpha }\\ +V\sum_{i}\left(c_{iA}^{†}c_{iA}-c_{iB}^{†}c_{iB}\right)+\text{h}.\text{c}. \end{array}$$

**Fig. 4.**
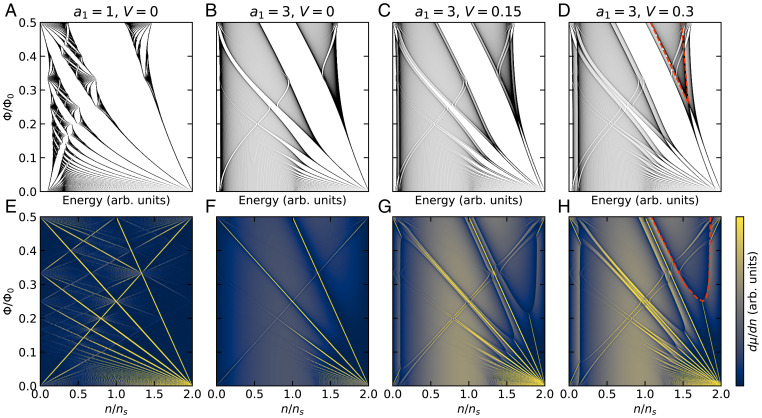
Replication of unusual magnetotransport features in Hofstadter’s butterfly. (*A*–*D*) Energy spectra for the indicated parameters for q=1,999, discussed in the main text. (*E*–*H*) Inverse density of states corresponding to spectra in *A*–*D*. For these plots, as well as their counterparts in Fig. 3, the density runs up to $n/n_{s}=2$ to account for the two fermion species. The dashed red line in *H* bounds one of the high density-of-states regions where the two butterflies overlap, which corresponds to where we see large magnetoresistance in transport.

In the following text and Figs. 3 and 4, we set $a_{2}=a_{3}=1$ and consider only constant *V*. If *V* is instead set proportional to *B*, to reflect Zeeman splitting of either spins or valleys, the phenomenology is not substantially changed. The specific values of *a*_1_ and *V* that we show are not motivated by any microscopic details of the system, but were instead chosen for their phenomenological match to the data. Fig. 4 shows spectra and the corresponding inverse density of states from the model of Eq. **2** for several values of *a*_1_ and *V*.

The spectrum for $a_{1}=a_{2}=a_{3}=1$ is identical to the classic isotropic butterfly, and the corresponding inverse density of states demonstrates clear Diophantine behavior (Fig. 4*E*). However, as there are two fermion species, each state is doubly degenerate and the gaps follow even-integer slopes only.

Anisotropy has previously been shown to smear out the energy levels and partially close the gaps in the spectrum (6, 8–10), which we reproduce by tuning *a*_1_ away from *a*_2_ and *a*_3_ (Fig. 4 *B* and *F*). Upon then introducing a small amount of *V*, a second butterfly pattern appears (Fig. 4*C*). At low fields and low densities the two butterflies are almost parallel and seldom overlap, and every integer filling of Landau levels gives a ground state with a gap for excitations. However, at higher fields and energies, the anisotropy-broadened butterflies overlap, and odd-integer Landau-level fillings have no gap to excitations. The even-integer Landau levels appear to split and bend in the same way as the measured Landau levels in our device, and the behavior at crossings of opposite-polarity Landau levels is also the same as in our device, as shown in Fig. 3 *C* and *D*. The shape of the split LLs is in rough agreement with our experiment for the range of parameters $1.5<a_{1}<4$ and 0.1<V<0.4. *SI Appendix* shows the behavior of the model outside of this parameter range and more fully explains the cause of the offsets in split LLs as they intersect.

While we cannot rule out the possibility of an alternative explanation, the fact that such complex behavior can be reproduced with a simple single-particle model is very encouraging. Even if a modified Hofstadter model is the correct description of the unusual magnetotransport in TBG, our particular choice of modifications is not the only one that could account for the data. For example, we found that a Hofstadter butterfly model with unit cell doubling plus anisotropy gives the same splitting, bending, and overlapping behavior. Further experiments will be needed to determine which modifications to a Hofstadter model are actually relevant to our TBG devices. With those caveats in mind, several features call for further examination:

First, what causes the striking magnetoresistance? We see very large magnetoresistance in the density-field region of our experimental fan diagram corresponding to where the two broadened butterflies overlap in our model, as indicated in Fig. 4. Although the phenomenological association is clear, it is not obvious to us why overlapping Landau levels should produce such prominent magnetoresistance. One might instead imagine that the magnetoresistance results from coexistence of charge carriers of both signs, since compensated semimetals show some of the strongest known near-quadratic magnetoresistance (44–46). This phenomenologically tempting explanation does not simply accord with the persistence of magnetoresistance over a broad gate voltage range (for a fuller discussion, see *SI Appendix*). Perhaps such coexistence could be related to proximity to van Hove singularities in the density of states (47, 48) (also see *SI Appendix*, Fig. S6 and associated discussion).

Second, is the smearing out of the energy spectrum caused by anisotropy, as we suspect? If so, what is the origin of the anisotropy? Some previous theoretical (49–54) and experimental (55–58) results suggest nematic order at a variety of filling factors within the lowest-energy moiré miniband manifold, both in TBG relatively close to the magic angle of 1.1^∘^ and in twisted double-bilayer graphene. One would expect that nematic order would accompany an anisotropic effective Hamiltonian. We do not believe that this explanation is relevant, simply because we see the unusual magnetotransport at large enough angles such that the electron interactions are too weak for nematic order to exist. Instead, we believe that uniaxial strain, either in one layer or in both, is a more likely explanation.

Landau fan diagrams have been a staple of electrical transport measurements for decades, because they give clear insight into the spectrum of electronic states and their filling. In this work, we have identified an entirely different confluence of phenomena in the fan diagram of a TBG device and have found, to our surprise, that this same combination emerges naturally from a single-particle Hamiltonian with anisotropic tunneling.

## Materials and Methods

### Device Fabrication.

We assembled our device using a tear-and-stack method. We first prepared a Poly(Bisphenol A carbonate) film stretched over a gel (Gel-Pak DGL-17-X8) and affixed it to a glass slide with double-sided tape. To start stacking, we picked up the top layer of hBN at 80 ${}^{{}^{\circ}}\text{C}$. We then used the edge of the hBN flake to pick up and tear the graphene at room temperature. The lower temperature compared to the other steps helps to prevent a common cause of stacking failure for us: graphene outside the region directly contacted by the hBN being picked up or dragged. In this step, we attempted to optically align a long, straight edge of the hBN to a similar edge of the graphene. We then rotated the remaining portion of the graphene flake by 1.2^∘^, picked it up at 80 ${}^{{}^{\circ}}\text{C}$, picked up the bottom hBN at 80 ${}^{{}^{\circ}}\text{C}$, and then finally picked up a flake of few-layer graphite at 80 ${}^{{}^{\circ}}\text{C}$ to form the back gate. We transferred the final stack at 150 ${}^{{}^{\circ}}\text{C}$ onto 300-nm-thick SiO_2_ on degenerately doped Si with prepatterned alignment marks.

We then used several iterations of standard e-beam lithography to define the Hall bar. We deposited a Ti/Au top gate, etched the Hall bar region using CHF_3_/O_2_ (50/5 sccm), and then deposited Cr/Au edge contacts.

### Transport Measurements.

All measurements in the main text were taken in a dilution refrigerator with a base temperature of 26 mK at the mixing chamber. The measurement lines include low-pass RF and discrete RC filters at the mixing chamber stage. We used a Stanford Research SR830 lock-in amplifier with a 1-GΩ bias resistor to source an alternating current of 1 nA at roughly 1 Hz. We measured differential voltage pairs with NF Corporation LI-75A voltage preamplifiers and SR830 lock-in amplifiers. We applied gate voltages using Yokogawa 7651 DC voltage sources. We held the Si back gate at a constant 30 V for all measurements to promote transparent contacts.

### Hofstadter’s Butterfly Calculation.

We calculated the butterfly spectra using standard numerical methods. Details can be found in *SI Appendix*.

## Supplementary Material

Supplementary File

## Data Availability

The data for this study along with all code used to generate spectra and figures have been deposited in the Stanford Digital Repository (https://doi.org/10.25740/tm725vs8229) (59).
