# Supplementary material for: Unusual magnetotransport in twisted bilayer graphene
Source: Proc Natl Acad Sci U S A. 2022 Apr 11;119(16):e2118482119. doi: 10.1073/pnas.2118482119 (PMC9169859; doi:10.1073/pnas.2118482119)
Supplement: Supplementary File [file pnas.2118482119.sapp.pdf]

PNAS
www.pnas.org

Supplementary Information for

Unusual magnetotransport in twisted bilayer graphene

Joe Finney, Aaron L. Sharpe, Eli J. Fox, Connie L. Hsueh, Daniel E. Parker, Matthew Yankowitz, Shaowen Chen, Kenji Watanabe, Takashi Taniguchi, Cory R. Dean, Ashvin Vishwanath, M. A. Kastner, and David Goldhaber-Gordon

David Goldhaber-Gordon.
E-mail: goldhaber-gordon@stanford.edu
M. A. Kastner.
E-mail: mkastner@mit.edu

This PDF file includes:

Supplementary text
Figs. S1 to S15 (not allowed for Brief Reports)
Table S1 (not allowed for Brief Reports)
SI References

Supporting Information Text

Twist angle determination

We initially tested the device in a variable temperature insert (VTI) system at 1.7 K using a homemade set of lock-in amplifiers that allowed us to measure every longitudinal and Hall voltage pair simultaneously. By sweeping density and field, we can see the overlap of the Landau levels originating at charge neutrality and full-filling, respectively. The spacing of these overlap points is constant in $1/B$ and is directly related to the area of the moiré unit cell, which allows for accurate calculation of the twist angle (1). In Fig S1c we show the twist angle variation across the device. The topmost contact pairs have a dramatically different twist angle than the rest. The rest of the contacts vary between 1.28° and 1.45°, with the majority near 1.36°. The contacts near 1.36° display the strongest magnetoresistance. All contact pairs with MR $>$ 10 show the unusual LL behavior discussed in the main text to some degree.

Effect of displacement field

The Si back gate is screened by the graphite back gate which extends only over the main conduction channel, but is not screened near the contacts. This allows us to set the carrier density near contacts to be high to encourage contact transparency. Meanwhile, the top and back gates allow us to individually tune density and displacement field. Unfortunately, when the back gate is near 0 V we find that our contact resistances dramatically increase in a magnetic field, leading to a loss of signal at lower temperatures. This happens regardless of the Si back gate voltage. Thus, for the measurements in the main text, we fix the back gate at 1.5 V and then sweep the top gate. This means that the measurements are not performed at a constant displacement field as we would prefer. Instead, they follow the black dashed line in Fig. S2c. As we will next discuss, varying the displacement field does not substantially change the phenomenology presented in the main text.

We calculate the displacement field as in reference (2). Fig. S2 shows the displacement field dependence of the contact pair from the main text at a few fixed magnetic field values. There is no apparent effect at 0 T. At 3 T, negative displacement field enhances the peak value of the magnetoresistance and widens the density extent of the magnetoresistance region. The resistivity at the charge neutrality point increases with increasing magnitude of displacement field, regardless of the polarity of this field. In Fig. S2c, we can clearly observe the split LLs within the magnetoresistance regions at 8 T as pairs of vertical lines, corresponding to constant density, independent of displacement field.

Behavior of other contact pairs

All measurements in the main text are of contact pair 16 - 17. We present Landau fan diagrams and gate maps of two other contact pairs in Fig. S3. In both cases, the basic phenomenology presented in the main text is reproduced. Contact pair 7 - 8 is very similar to 16 - 17. Contact pair 4 - 5 displays split LLs, though less clearly defined.

In both of these cases, there are oscillations as we tune the displacement field at certain densities. We do not have a satisfactory explanation for this observation, however we note that they appear to be more closely related to the value of the back gate voltage rather than the displacement field (this is reflected in their downward slope in Fig. S3d; lines of constant gate voltage are sloped since a transformation has been applied to make the axes of that figure n and D). Just as the back gate gates the moire channel, the moire channel gates the back gate, so band filling in the 4-5 layer thick back gate is determined primarily by applied back gate voltage. Empirically, at zero back gate voltage, contacts to the moire channel become high resistance, perhaps reflecting low density of carriers in the back gate. At high magnetic field as in Fig. S3c, carrier density in the back gate might be substantially and nonmonotonically modulated with back gate voltage. One way this could affect electronic properties of the moire channel is through changes in screening, given that the lower hBN layer is only about 13 nm thick, comparable with the moiré wavelength. Regardless of the reason for the back-gate-specific effect, the choice of fixing back gate voltage and sweeping top gate for the figure in the main text may actually be superior to holding the displacement field fixed, since to do that would require us to vary the back gate at the same time.

Additional phenomenology near charge neutrality

Fig. S4 shows the longitudinal magnetotransport at low field and low density for contact pair 7 - 8. The other two contact pairs behave in a similar manner. Some of the Landau level gaps disappear and then reappear as the field is increased. This same behavior can be seen in the model, in Fig. 4g and h from the main text, which is reproduced in S4b: there we can see the Landau levels from the two butterflies intersect at low field, leading to a gradual disappearance and then reappearance of the gap.

Unusual horizontal lines (constant field) appear in between many of the Landau levels. These lines appear to take steps upon crossing Landau levels. These are seen to some extent in all three contact pairs, and are also seen near full-filling and emptying ($n/ns = \pm 4$). Within a simple Hofstadter model with only one fermion species but additionally with a next-neighbor hopping term, these horizontal lines can be qualitatively reproduced (Fig S4c and d): B-field-periodic modulation in the width of the Landau levels causes horizontal lines of reduced resistivity corresponding to fields where the Landau level is narrower and thus has increased DOS. The parameter tuning that gives such modulation is independent of that needed for the phenomenology highlighted in the main text (one can get either, both, or neither). In a more physically realistic model of the moire this phenomenon may be more firmly linked to the rest.

Superconductivity at 1.33°

Contact pairs 13 - 14 ($\theta = 1.33°$) and to a lesser extent 3 - 4 ($\theta = 1.35°$) display evidence of superconductivity near half filling of holes. We show measurements from 13 - 14 in Fig. S5. To our knowledge, superconductivity has not been previously reported for a sample so far above the magic angle, and in our measurements the superconductivity is significantly weaker than that in samples near the magic angle, as demonstrated by its low critical temperature of 150 mK, low critical field of 3 mT, and low critical current of 12 nA. Contact pair 3-4 does not have any significant magnetoresistance, and 13-14 has relatively low magnetoresistance (MR ratio reaches ~ 15).

Low-field Hall effect

Fig. S6 shows the Hall density for contact pair 13 - 3 ($\theta = 1.33°$). We do not observe a resetting of the Hall density at integer filling factors, as is seen in samples near the magic angle. Instead, roughly in the center of each magnetoresistance region, the Hall density diverges and then changes sign. At these points, the Fermi level crosses a van Hove singularity(3). All contact pairs in the device display similar behavior, as does D34 in the supplement. In these magnetoresistance regions, the Hall slope becomes nonlinear at higher fields. The Hall density shown here is only based on the low-field slope.

Temperature dependence of the magnetoresistance

Fig. S7 shows the behavior of the magnetoresistance as a function of temperature, qualitatively similar to that seen in WTe_2 (4, 5), Cd_3As_2 (6), and other compensated semimetals. In these materials, it is understood that the Hall voltage from one carrier type cancels that from the other type, leading to circular charge carrier trajectories and thus reduced carrier diffusion and positive magnetoresistance.

If the magnetoresistance in our device were the result of a compensated semimetal, we would not expect the magnetoresistance to be so consistent over such a large range of gate-tuned total density. Also, we would expect to see sets of Landau levels originating at the edges of each isolated pocket in the Fermi surface, whereas we only see them originating at charge neutrality and moiré band edges. The split Landau levels have the same periodicity in $1/B$ as each other (Fig. S8), so they correspond to the same density offset from their respective band edge. This has motivated us to seek an alternative explanation.

Hofstadter calculation details

We start with the Hamiltonian from Eq. 2 of the main text:

$$H = \sum_{\alpha \in \{A,B\}} \sum_{\langle i,j \rangle} a_{ij} c^\dagger_{i\alpha} c_{j\alpha} + V \sum_i \left(c^\dagger_{iA} c_{iA} - c^\dagger_{iB} c_{iB} \right) + \text{h.c.} \qquad [1]$$

where i and j are lattice sites of a triangular lattice with lattice vectors (0, 1), ($\sqrt{3}$/2, 1/2), and ($\sqrt{3}$/2, -1/2), and A and B are the two fermion species. In the Landau gauge, the vector potential is $\mathbf{A} = (0, Bx, 0)$, and the hopping terms pick up a Peierl's phase of $e/\hbar \int \mathbf{A} \cdot d\mathbf{r} = 2\pi\phi x$ for the first lattice vector and $\pi\phi(x \pm 1/2)$ for the remaining two. Considering only rational values $\phi = p/q$ for coprime p and q, the Peierl's phase repeats after q hops in the x direction, and so we define the magnetic Brillouin zone (MBZ, not to be confused with mini or moiré BZ) as $0 < k_x < 2\pi/q$ and $0 < k_y < 2\pi$. In principle, we should vary k_x and k_y throughout the MBZ to extract the full energy spectrum. We will only concern ourselves with large values of q, for which the width of each subband becomes extremely small, so we only solve for $k_x = k_y = 0$.

The Peierl's substitution removes all hopping terms in the y direction from the Hamiltonian, so eigenstates should be plane waves in y, which leads to the Hamiltonian

$$H = \sum_{\alpha \in \{A,B\}} \sum_{m=1}^{q} \left[a_1 \cos(2\pi\phi m) c^\dagger_{m\alpha} c_{m\alpha} + a_2 \cos(\pi\phi(m+1/2)) c^\dagger_{m+1,\alpha} c_{m,\alpha} + a_3 \cos(\pi\phi(m-1/2)) c^\dagger_{m-1,\alpha} c_{m,\alpha} \right] + V \sum_{m=1}^{q} \left(c^\dagger_{mA} c_{mA} - c^\dagger_{mB} c_{mB} \right) + \text{h.c.}$$

As this is a finite 1D Hamiltonian, it is now straightforward to numerically compute its eigenvalues. The most convenient way to compute the spectrum over a large range of magnetic field values is to set q to some large prime number (typically thousands, depending on the available computation power) and then solve the above equation for p from 1 to $q-1$.

As the gaps in the spectrum appear as extremely fine, bright lines in inverse density of states, they are susceptible to aliasing artifacts when the images are downsampled. To suppress these artifacts, we apply a fine gaussian filter ($\sigma \sim 1/q$) to these plots. This does not affect the phenomenology.

We show the behavior of the split LLs for a range of hopping parameters in Fig. S9. We have also made calculations on square and honeycomb lattices with anisotropy and two fermion species, and have verified that the behavior is qualitatively similar to that on the triangular lattice. Without fine tuning we can replicate the disappearance of odd LLs, the split even LLs, and the split intersection behavior. We also checked that the same behaviors replicate for $V \propto B$ (Fig. S10). Though these are not microscopically faithful models for TBG, many features of the Hofstadter problem are set by the topology of the bands alone and thus should be only weakly model-dependent.

Split Landau level intersection behavior in the model

The complicated split LL intersection behavior from our model is not difficult to understand, however it is somewhat intricate. We begin by noting that many of the gaps in Hofstadter's butterfly take horizontal steps—a discontinuity in energy at a given magnetic field—when they cross gaps moving the other direction (Fig. S11 a and b). When the second butterfly appears out of the first (Fig. S11c and d), it is shifted horizontally in energy. We then effectively have two Xs next to each other. When two upper or two lower split LLs overlap, the system is simultaneously in the gap of both butterflies, and so we see the average (s, t) of the two intersections. When an upper meets a lower split LL, we are seeing the intersection of LLs within a single butterfly. Therefore, it happens at the appropriate field for that intersection. As there is a background DOS from the other butterfly, we see the horizontal step in energy that is present even in undoubled lattices.

Comparison to additional devices

We present measurements from three additional devices with twist angles 1.59° (D34, Fig. S12), 1.52° (D25, Fig. S13), and 1.70° (Fr, Fig. S14). Unlike the device from the main text as well as Fr, D34 and D25 have dual graphite gates, and none of the three have purposefully-aligned hBN. D34 and D25 were measured in a four-probe configuration at 300 mK with a 10 nA AC current bias at 17.7 Hz. For more details, see ref. 31 and its supplementary text. Fr was measured in the same system as the device from the main text.

D34 and D25 show the same generic Landau level progression as the device from the main text, with dominant Landau levels $\nu = \pm 4, \pm 8, \pm 12, \ldots$. Both devices have regions of positive magnetoresistance, reaching ratios of ~ 30 in D34 and ~ 10 in D25 at 3 T. The magnetoresistance is significantly weaker than that of the device in the main text, and it is also not a clean quadratic at low field. While D25 does have an intricate and beautiful fan diagram, it does not display split Landau levels. D34 shows Landau level splitting comparable to that in the device from the main text for filling fractions $\nu = \pm 12, \pm 16, \pm 20, \ldots$. Although much of the fine detail of the split Landau levels appears washed out, the intersecting behavior from the text is also visible for some pairs of overlapping levels.

Fr's LLs (originating at CNP) $\nu = -8, -16, \ldots$, as well as $\nu = 20, 28, \ldots$ are not visible. Instead, we see only split LLs around where they should be. We additionally tested the effect of a 4 T in-plane field on Fr, and found no effect (Fig. S15).

We summarize the magnetotransport phenomenology for the four devices and their separate contact pairs in Table S1.

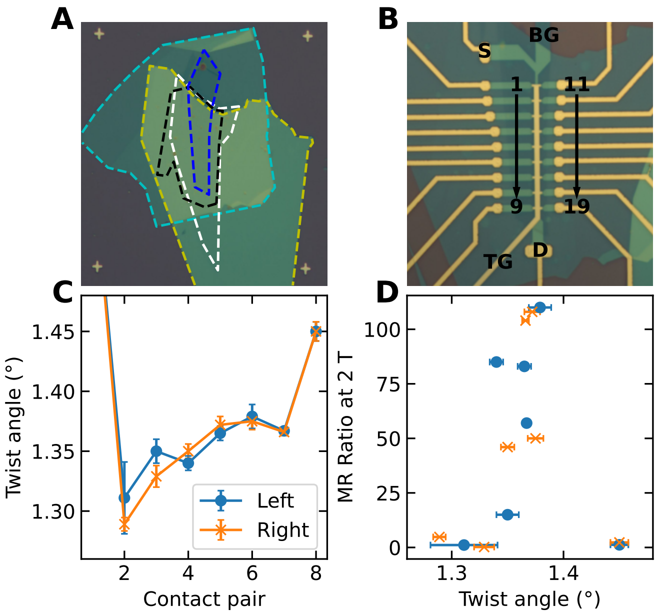

Fig. S1. Twist angle variation throughout the device. (**A**) Device layout prior to lithography with separate layers outlined. From top to bottom we have top hBN (yellow, 20 nm thick), top graphene (white), bottom graphene (black), bottom hBN (cyan, 13 nm thick), and graphite back gate (blue). The plus-shaped alignment marks in the corners are 100 µm apart. (**B**) Finished device layout after lithography. The labels are for the current source contact (S), current drain contact (D), back gate (BG), top gate (TG), Hall voltage probes on the left (1-9), and on the right (11-19). (**C**) Twist angle variation along the device as measured by the longitudinal magnetotransport at high density and field. The first set of contact pairs on either side is at 1.9°. (**D**) Magnetoresistance ratio at 2 T at fixed density 2.3×10^{12} cm^{-2} at 1.7 K. The magnetoresistance for the contact pairs at 1.9° (not shown) are 0.8 and 0.3 on the left and right respectively.

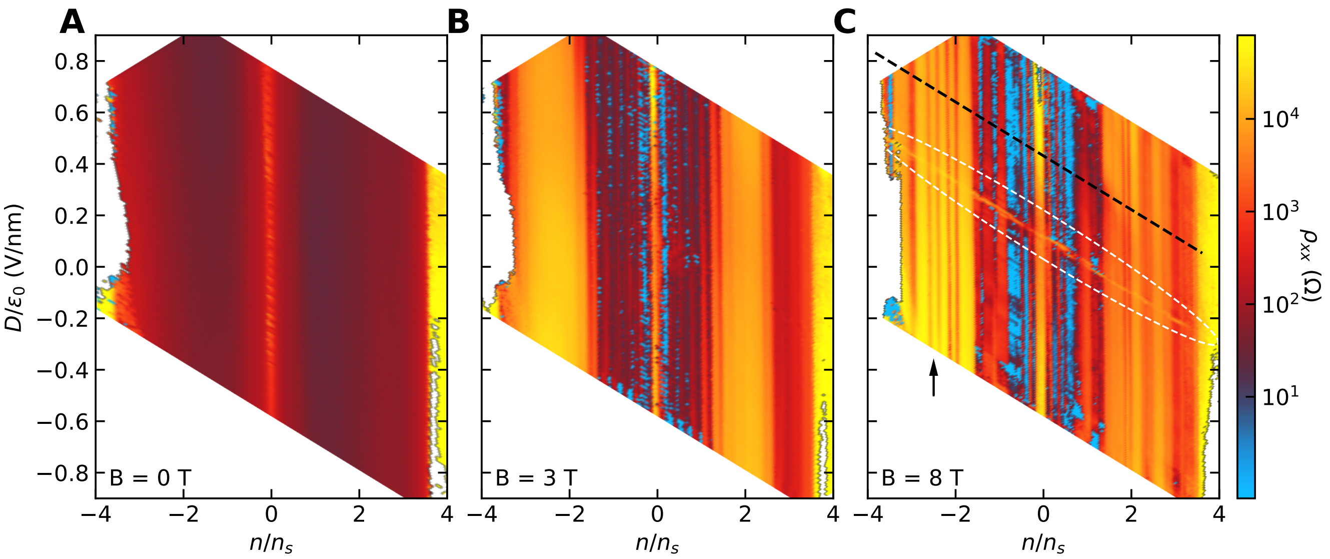

Fig. S2. Effect of electric displacement field. (**A**) Gate maps at 0 field, (**B**) at 3 T, and (**C**) at 8 T. The black dashed line is the cut used for the figures in the main text, while the white ellipse indicates the loss of signal when the back gate is fixed at 0 V. The arrow indicates one pair of split LLs, which appear as pairs of vertical lines within the magnetoresistance regions. That they are vertical indicates that they do not vary with displacement field.

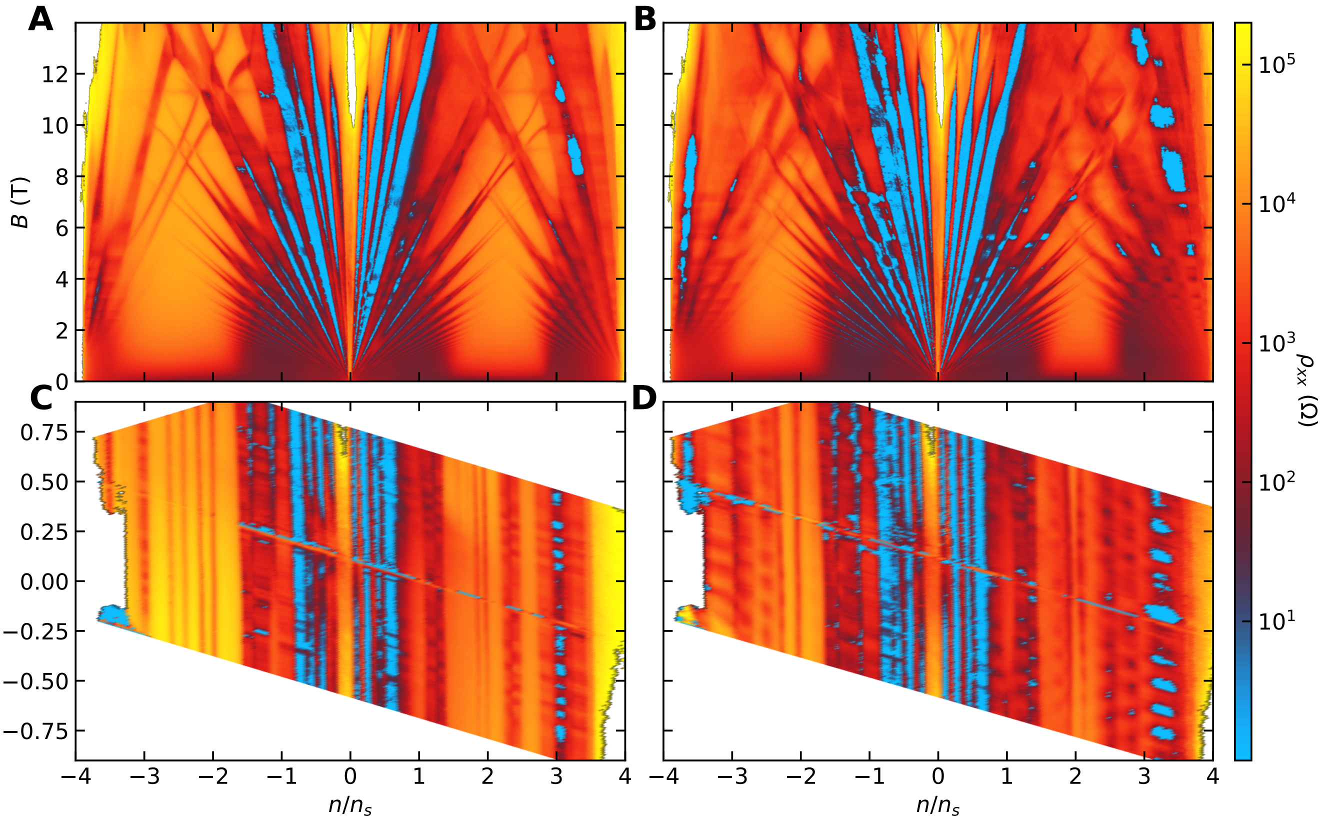

Fig. S3. Similar behavior in other contact pairs. (**A**) Fan diagram for contact pair 7 - 8 and (**B**) 4 - 5. (**C**) Gate map at 8 T for contact pair 7 - 8 and (**D**) 4 - 5.

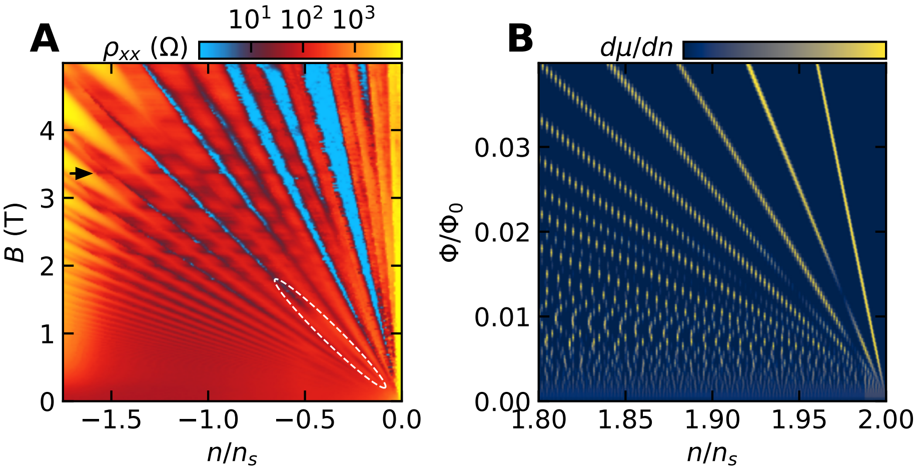

Fig. S4. Additional phenomenology captured by the model. (**A**) Magnetotransport for contact pair 7 - 8 at low field and low density. The dashed circle indicates the feature reproduced in simulation in panel B, and the arrow indicates one of the horizontal lines for which we have no satisfactory explanation. (**B**) Inverse density of states near a band edge at low field for $a_1 = 3$, $V = 0.3$, the same parameters as in Fig 4h. This is taken at $q = 1999$.

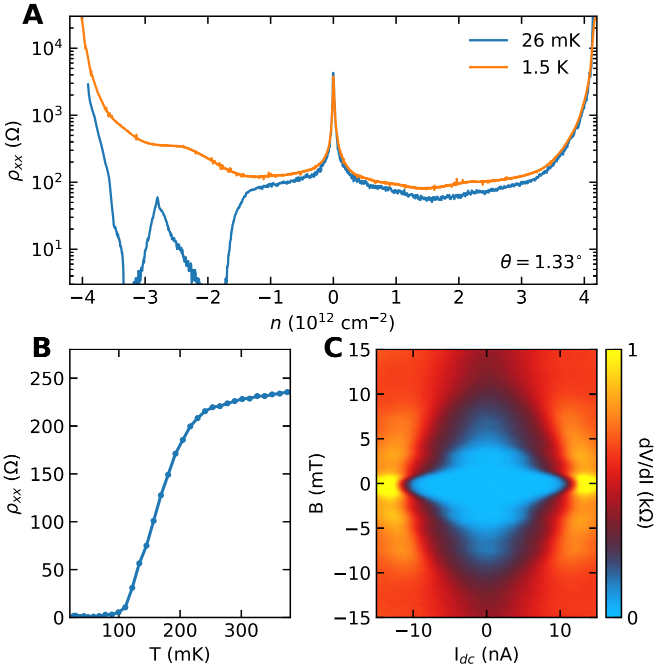

Fig. S5. Superconductivity at 1.33°. (**A**) Resistivity of contact pair 13 - 14 as a function of density at the indicated temperatures. (**B**) Temperature dependence of the superconductivity at fixed density at $n = -3.2 \times 10^{12}$ cm^{-2}. (**C**) Differential resistance as a function of DC bias current and field demonstrating weak Fraunhofer-like behavior at $n = -3.2 \times 10^{12}$ cm^{-2}. We have removed a small flux jump around $B = -7$ mT in preparing this figure.

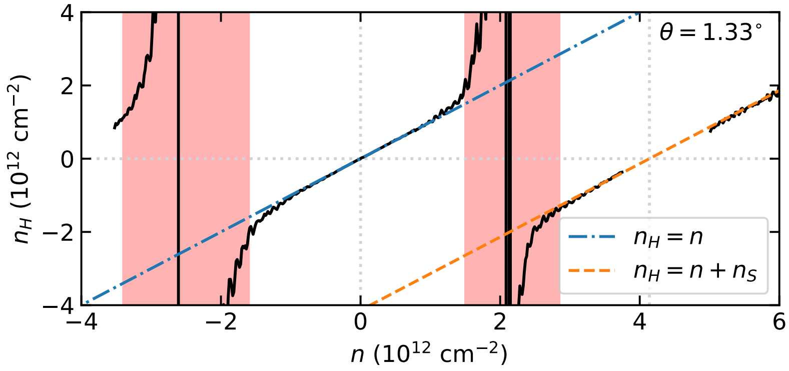

Fig. S6. Low-field Hall effect. The Hall density for contact pair 13 - 3, as measured between -0.5 and 0.5 T. The regions of large magnetoresistance are highlighted in red. Near $n/n_s = \pm 4$, we lose our measurement signal due to the increased sample resistance.

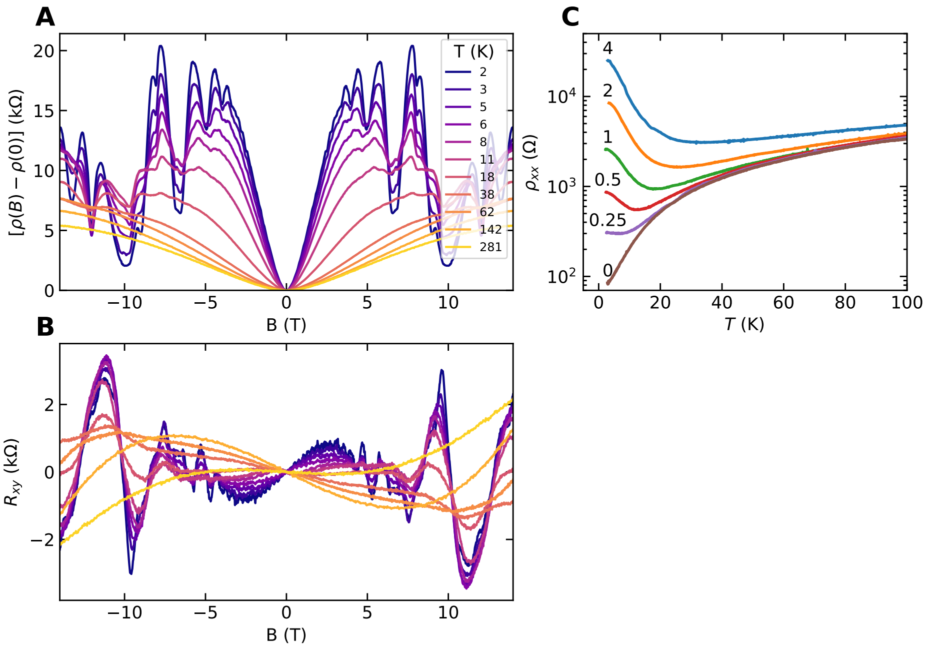

Fig. S7. Temperature dependence of large magnetoresistance. (**A**) Temperature dependence of longitudinal resistivity (contact pair 16 - 17, symmetrized) and (**B**) Hall resistance (contact pair 6 - 16, antisymmetrized) at $n/n_s = 2.5$. (**C**) Temperature dependence of longitudinal resistivity at fixed fields at $n/n_s = -2.8$.

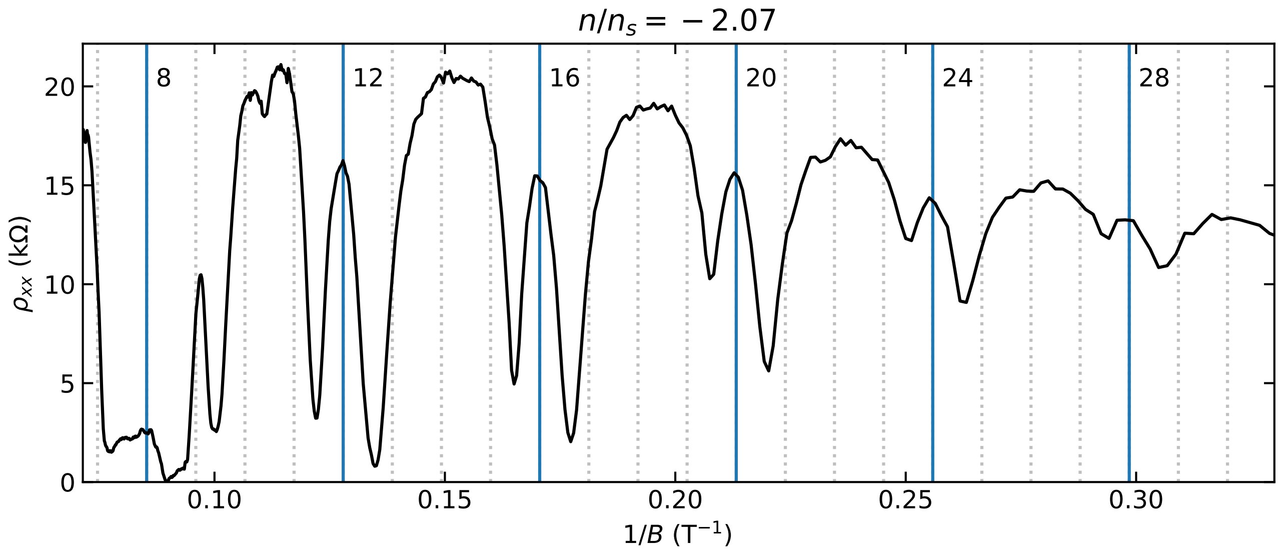

Fig. S8. Split Landau levels as a function of $\mathbf{1/B}$. Longitudinal resistivity at fixed density within the magnetoresistance region as a function of $1/B$. The vertical lines indicate expected Landau level filling factors originating from charge neutrality. Note that the split LLs correspond to roughly $\nu = 11.4, 12.6, 15.4, 16.6, \ldots$ ($\nu = 8$ has extra features originating from $n/n_s = -4$). That the split LLs have the same period in $1/B$ indicates that they correspond to the same density offset from charge neutrality and are not a product of multiple Fermi surfaces or band reorganization.

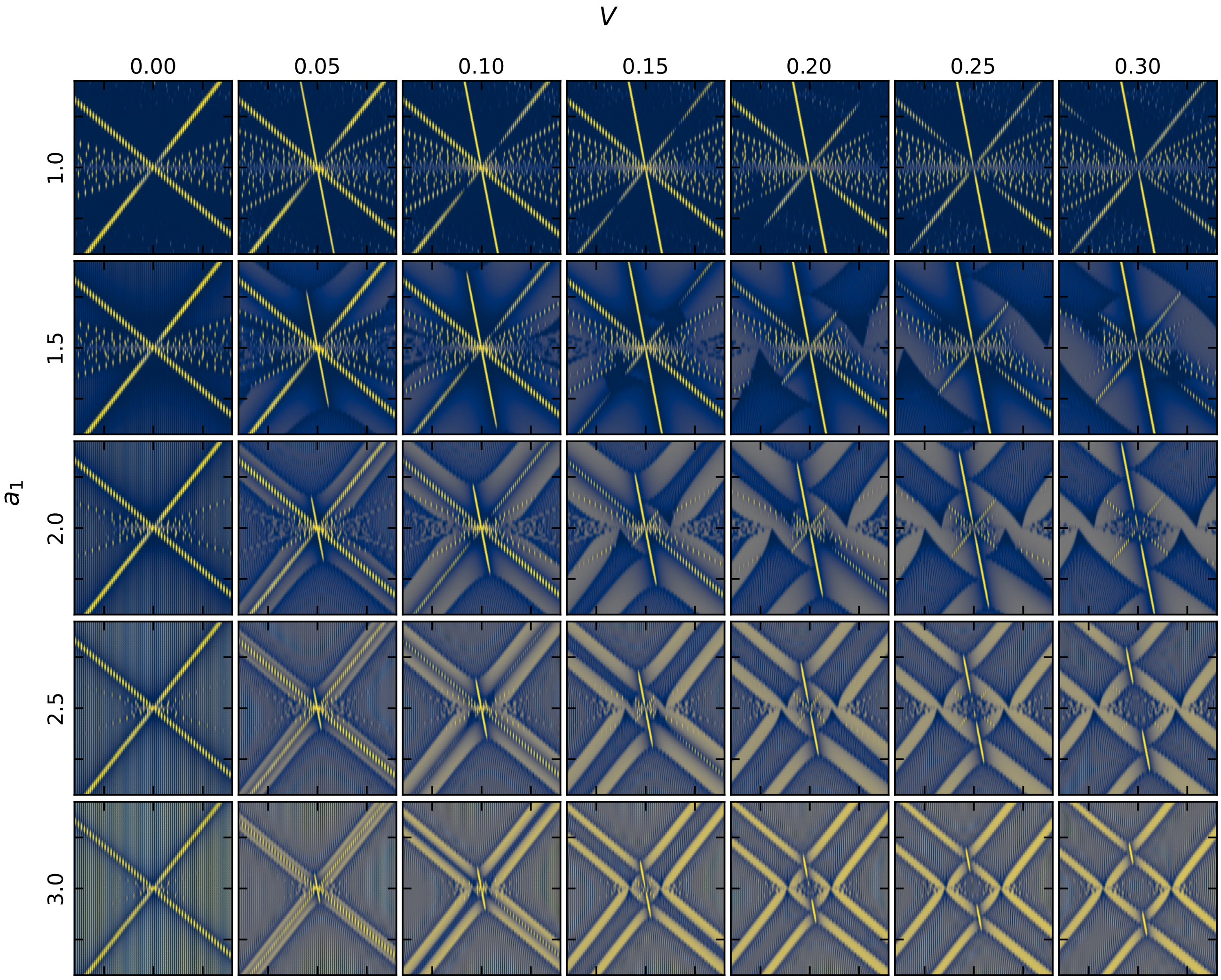

Fig. S9. Model behavior as a function of a_1 and V. Each plot shows the same region of (n, B) as in Fig. 3c of the main text, taken at $q = 1999$ and $a_2 = a_3 = 1$. The combination of experimentally observed qualitative phenomenology is reproduced by the model not just at a fine-tuned value of the parameters a_1 and V but rather for ranges of parameters a_1 and V such that $2.5 < a_y < 4$ and $0.1 < V < 0.4$. The ranges are comparable for different (n, B). For parameter values that show clear split LLs, increasing V increases the distance between the split LLs (which goes to zero when $V = 0$), and increasing a_1 decreases their width. Increasing a_1 beyond the stated range suppresses the discontinuities at LL crossings, as a direct consequence of the decreasing width of the LLs: compare (a_y, V) = (2.5, 0.3) to (3, 0.3).

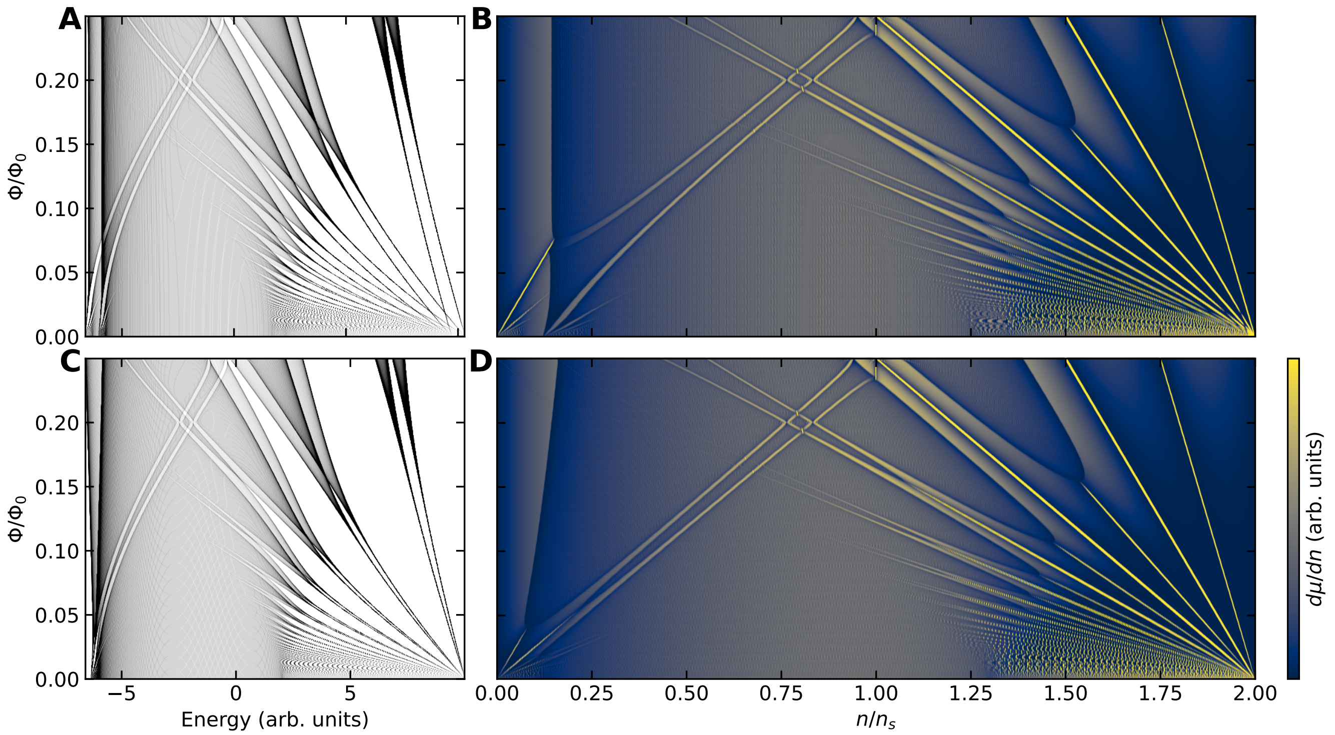

Fig. S10. Comparison of Zeeman-like splitting to fixed splitting. Energy spectra ($q = 1499$) and inverse density of states for $a_x = 1$, $a_y = 2$, and (**A-B**) $V = 0.2$, (**C-D**) $V = \Phi/\Phi_0$. The two models produce qualitatively very similar phenomenology. There is one notable but subtle differences: At low fields near charge neutrality and full filling, the constant-V Landau levels from the two butterflies cross each other, whereas in the Zeeman-like model the two butterflies are not split at low field and thus cannot cross at low field. These crossings lead in the constant V model to the disappearance and reappearance of gaps along the density/field trajectory of a given Landau level, as in Fig. S4b. This phenomenon seems to happen in experimental plot S13b at ($n = 0.9 \times 10^{12}$ cm^{-2}, $B = 3$ T) and less prominently in measurements on at least three contact pairs of the device from the main text that show LL splitting. Though this may favor the constant V model as a description of the experiment, more measurements would be needed to definitively support that assignment.

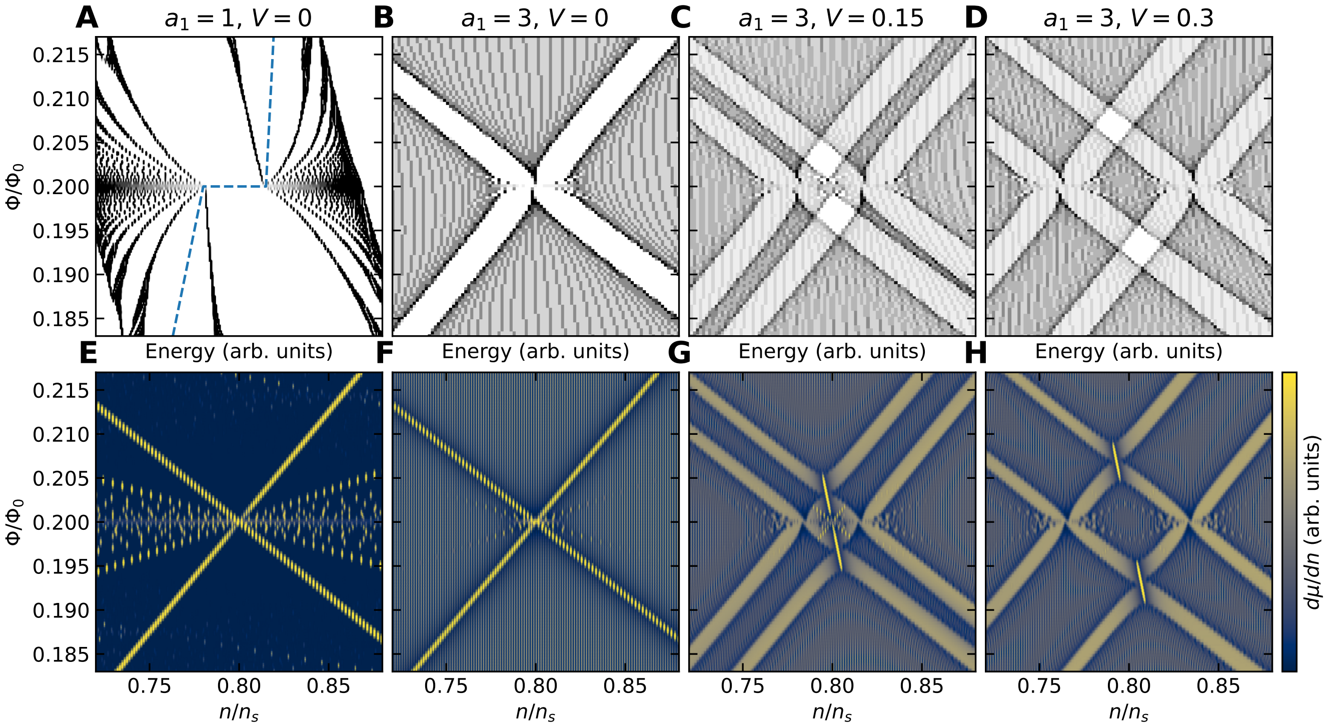

Fig. S11. Split LL detail in the model. (**A-D**) Energy spectra for the indicated parameters for $q = 2999$, zoomed in to the intersection of the LLs $s, t = 4, -6$ (gap indicated with the dashed line) and $-2, 4$. (**E-H**) Associated inverse DOS for the respective spectra. Panel **H** shows the same parameters as Fig. 3c.

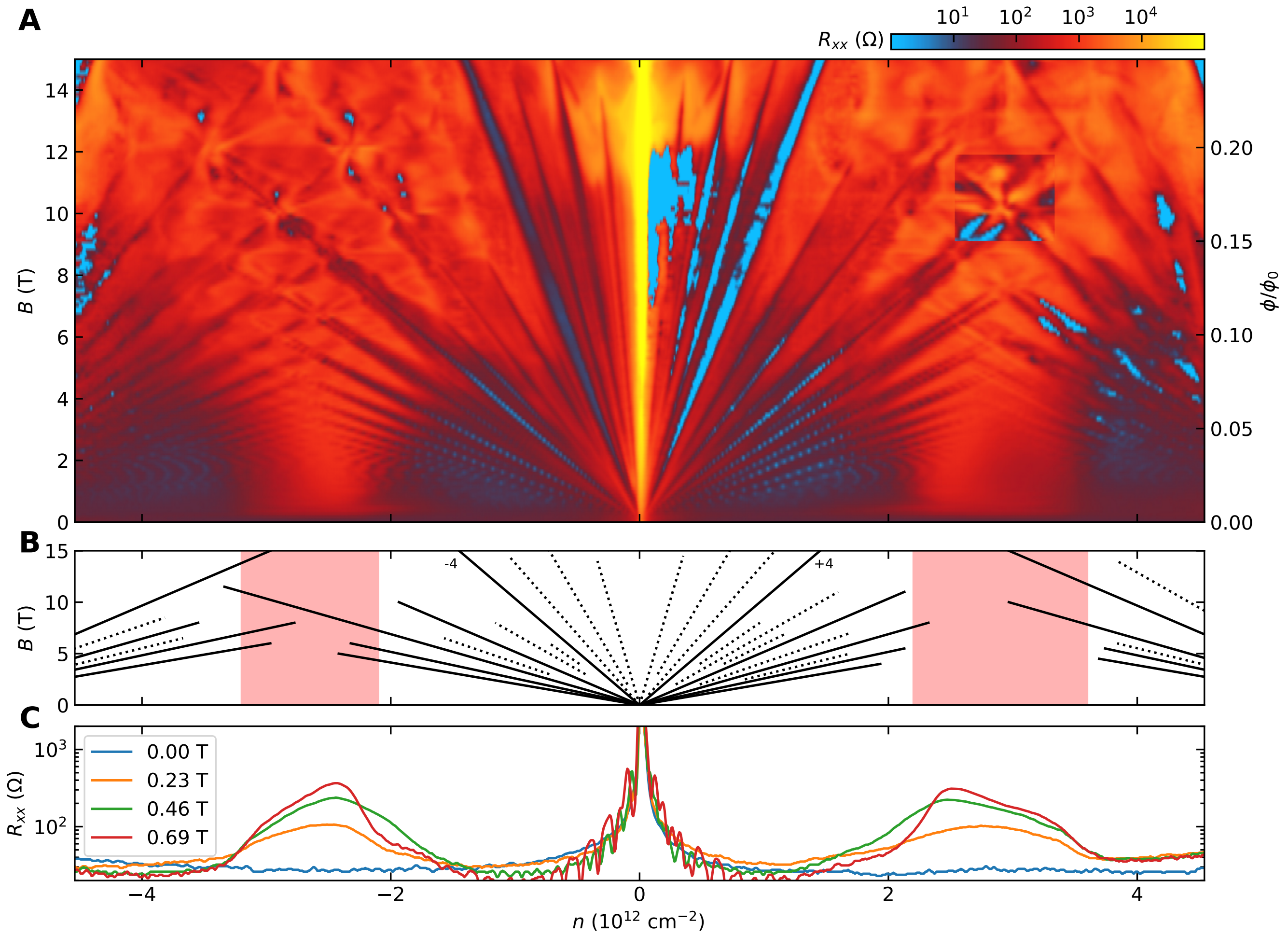

Fig. S12. Replication of split LLs in a second device. (**A**) Longitudinal resistance vs density and perpendicular magnetic field for device D34 ($\theta = 1.59°$). The window with increased contrast (color bar does not apply) highlights the intersection of $\nu = 12$ from charge neutrality and $\nu = -12$ from full filling. There is a faint vertical line, corresponding to the average of the slopes of the two Landau levels. (**B**) Schematic fan diagram corresponding to (A), with the same legend as Fig. 2 in the main text. (**C**) Line cuts from panel A at the indicated fields, showing regions of magnetoresistance with location and shape similar to those in Fig. 1b.

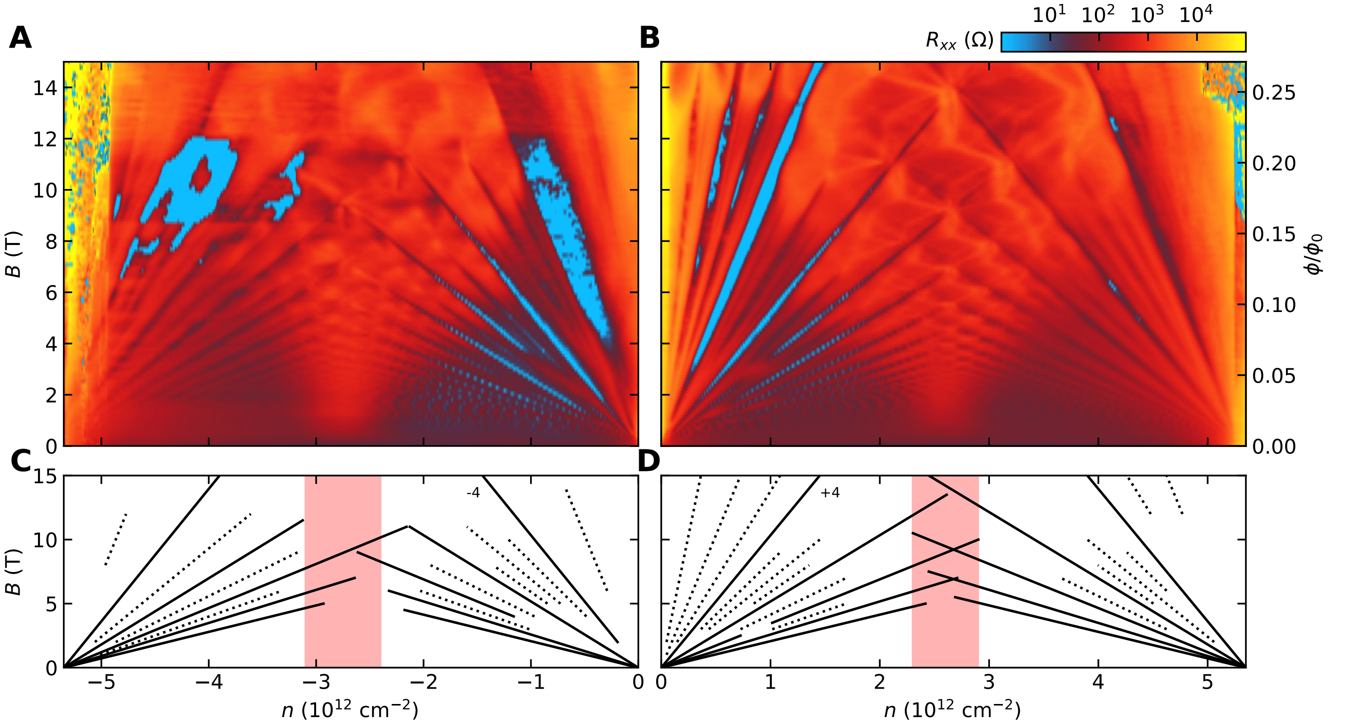

Fig. S13. Lack of split LLs in a third device similarly far from magic angle. Longitudinal resistance vs density and perpendicular magnetic field for device D25 ($\theta = 1.52°$) for holes (**A**) and electrons (**B**). The measurements for electrons and holes are taken in different contact pairs and at different Si gate voltages. (**C-D**) Schematic fan diagram corresponding to (A) and (B) respectively, with the same legend as Fig. 2 in the main text.

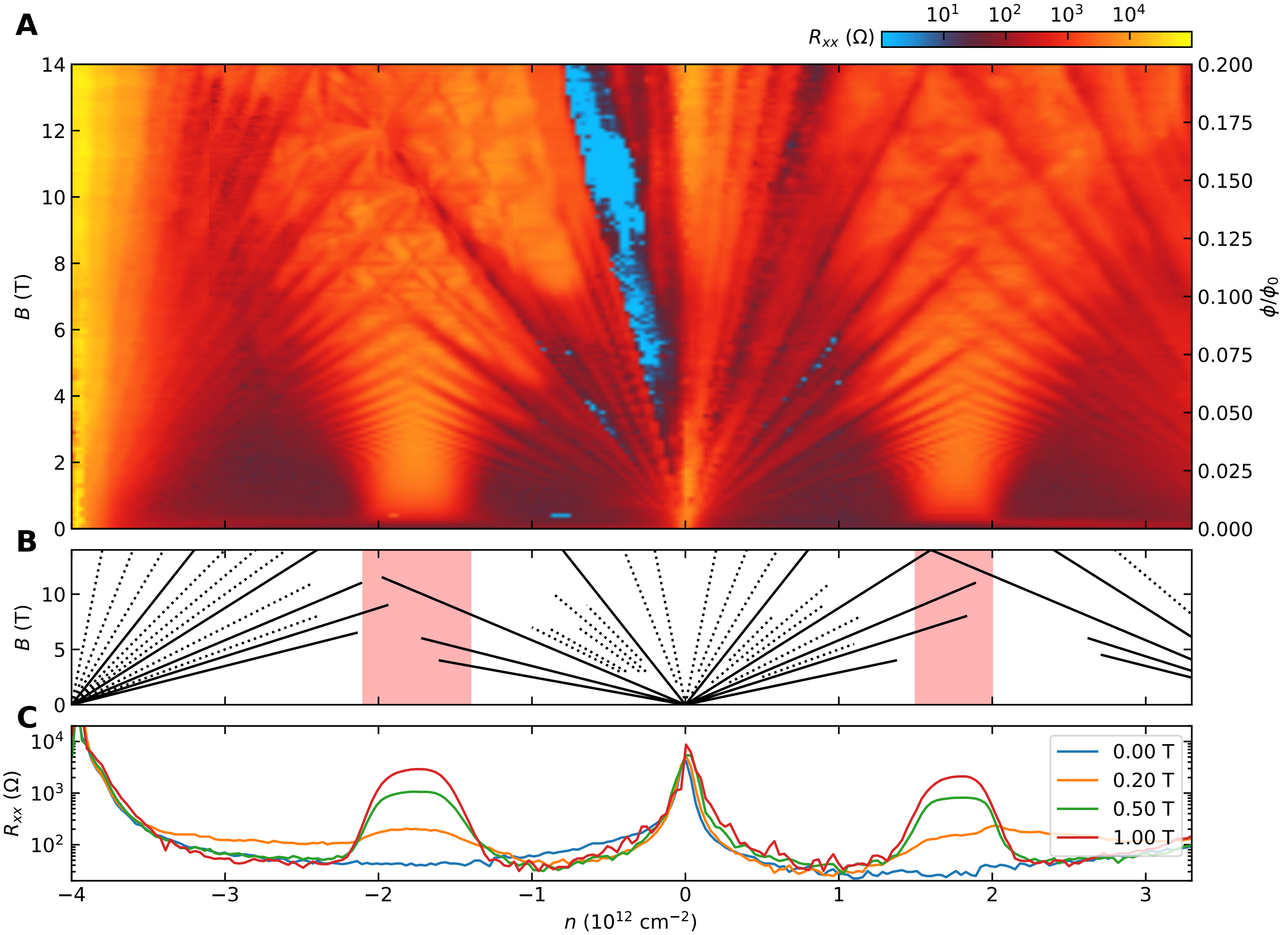

Fig. S14. Split LLs in a third device. (**A**) Longitudinal resistance vs density and perpendicular magnetic field for device Fr ($\theta = 1.70°$). (**B**) Schematic fan diagram corresponding to (A), with the same legend as Fig. 2 in the main text. (**C**) Line cuts from panel A at the indicated fields, showing regions of magnetoresistance with location and shape similar to those in Fig. 1b.

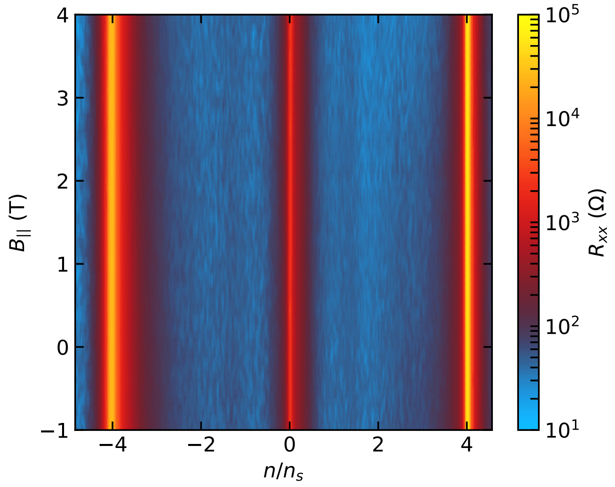

Fig. S15. Effect of in-plane field on device Fr. Longitudinal resistance vs density and parallel magnetic field for device Fr ($\theta = 1.70°$), measured at 1.5 K. From the Hall slope we calculated that the sample tilt angle was roughly 0.7° away from perpendicular.

Table S1. Summary of the separate contact pairs and devices presented in the text. The columns are device and contact pair, temperature at which the measurements were taken, twist angle, saturation field, largest magnetoresistance ratio, and whether or not we see clear split Landau levels. Though measurements were made at different temperatures for different devices, all those temperatures were well below 1K, whereas MR is not strongly temperature-dependent below 3K (cf. Fig. S7.) All rows except "D25 electrons" refer to the magnetoresistance region on the hole side. For all rows, the resistivity at zero field in the magnetoresistance regime is several tens of Ohms. B_{sat} is a very rough estimate of the lowest field at which the MR is within a few % of its maximum, MR$_{max}$. These values are hard to quantify to better accuracy than $\sim 10\%$ because quantum oscillations tend to become comparable in amplitude with the magnetoresistance at roughly the field at which it appears to saturate.

Device	Contact pair	T (mK)	θ (°)	B_{sat} (T)	MR$_{max}$	Split LLs?
NH	16 - 17	26	1.38	8	340	Very clear
NH	7 - 8	26	1.37	8	280	Very clear
NH	4 - 5	26	1.35	5	210	Clear
D34		300	1.59	3	30	Clear
D25	Holes	300	1.52	3	10	Absent
D25	Electrons	300	1.52	3	10	Absent
Fr		30	1.70	2	160	Clear

References

1. Y Cao, et al., Correlated insulator behaviour at half-filling in magic-angle graphene superlattices. *Nature* **556**, 80–84 (2018).
2. AL Sharpe, et al., Emergent ferromagnetism near three-quarters filling in twisted bilayer graphene. *Science* **365**, 605–608 (2019).
3. Y Kim, et al., Charge inversion and topological phase transition at a twist angle induced van hove singularity of bilayer graphene. *Nano Lett.* **16**, 5053–5059 (2016) PMID: 27387484.
4. MN Ali, et al., Large, non-saturating magnetoresistance in wte2. *Nature* **514**, 205–208 (2014).
5. V Fatemi, et al., Magnetoresistance and quantum oscillations of an electrostatically tuned semimetal-to-metal transition in ultrathin WTe 2. *Phys. Rev. B* **95**, 041410 (2017).
6. T Liang, et al., Ultrahigh mobility and giant magnetoresistance in the Dirac semimetal Cd3As2. *Nat. Mater.* **14**, 280–284 (2015).
